# Associations Between Self-Esteem and Postpartum Depression and Anxiety: A Cross-Sectional Study Using the Rosenberg Scale in Romanian Women

**DOI:** 10.3390/jcm15031135

**Published:** 2026-02-01

**Authors:** Nadica Motofelea, Costin Berceanu, Florica Voita-Mekeres, Radu Galis, Florin Adrian Szasz, Alexandru Catalin Motofelea, Teodora Hoinoiu, Ion Papava, Flavius Olaru, Daniel Viorel Soava, Maja Vilibić, Ionela-Florica Tamasan, Alexandru Blidisel, Adrian Carabineanu, Dan-Bogdan Navolan

**Affiliations:** 1 Doctoral School, “Victor Babes” University of Medicine and Pharmacy Timisoara, 300041 Timisoara, Romania; nadica.motofelea@umft.ro; 2 Department of Obstetrics and Gynecology, “Victor Babes” University of Medicine and Pharmacy Timisoara, Eftimie Murgu Square No. 2, 300041 Timisoara, Romania; olaru.flavius@umft.ro (F.O.); danisoava99@gmail.com (D.V.S.); navolan@umft.ro (D.-B.N.); 3 Department of Clinical Practical Skills, “Victor Babes” University of Medicine and Pharmacy Timisoara, 300041 Timisoara, Romania; tstoichitoiu@umft.ro; 4 Department of Obstetrics Gynecology, University of Medicine and Pharmacy, 200349 Craiova, Romania; 5 Department of Morphological Disciplines, Faculty of Medicine and Pharmacy, Oradea University, 410087 Oradea, Romania; 6 Department of Medical Sciences, Faculty of Medicine and Pharmacy, Oradea University, 410087 Oradea, Romania; rgalis@uoradea.ro; 7 Department of Obstetrics Gynecology, Faculty of Medicine and Pharmacy, Oradea University, 410087 Oradea, Romania; fszasz@uoradea.ro; 8 Center of Molecular Research in Nephrology and Vascular Disease, “Victor Babes” University of Medicine and Pharmacy Timisoara, Eftimie Murgu Square No. 2, 300041 Timisoara, Romania; alexandru.motofelea@umft.ro; 9 Center for Advanced Research in Cardiovascular Pathology and Hemostaseology, “Victor Babes” University of Medicine and Pharmacy Timisoara, 300041 Timisoara, Romania; 10 Department of Psychiatry, “Victor Babes” University of Medicine and Pharmacy Timisoara, 300041 Timisoara, Romania; papava.ion@umft.ro; 11 Department of Psychiatry, Sestre Milosrdnice University Hospital Center, 10000 Zagreb, Croatia; maja.vilibic@gmail.com; 12 School of Medicine, Catholic University of Croatia, 10000 Zagreb, Croatia; 13 Department XI: Pediatrics, “Victor Babes” University of Medicine and Pharmacy Timisoara, 300041 Timisoara, Romania; tamasan.ionela@umft.ro; 14 Department IX, General Surgery, Hepato-Bilio-Pancreatic Surgery Center, “Victor Babeș” University of Medicine and Pharmacy Timisoara, 300041 Timisoara, Romania; blidy@umft.ro; 15 Department IX Surgery I, Second Discipline of Surgical Semiology, “Victor Babes” University of Medicine and Pharmacy Timisoara, 300041 Timisoara, Romania; carabineanu.adrian@umft.ro

**Keywords:** self-esteem, postpartum depression, anxiety, Rosenberg Self-Esteem Scale, EPDS, GAD-7, Romania

## Abstract

**Background**: Postpartum depression and anxiety are common in the perinatal period and can adversely affect maternal functioning and infant outcomes. Self-esteem is a relevant psychosocial factor, yet evidence from Eastern Europe remains limited. **Objectives**: To describe self-esteem levels among postpartum Romanian women, examine correlational associations between self-esteem and postpartum depression/anxiety symptoms, and assess whether these associations persist after adjustment for sociodemographic and clinical covariates, across two maternity centers in a cross-sectional design. **Methods**: This cross-sectional study included 201 postpartum women recruited consecutively during their initial postpartum hospitalization from two public maternity hospitals in Western Romania (Bihor, n = 100; Timiș, n = 101) during 2024–2025. Participants completed the Rosenberg Self-Esteem Scale (RSES), Edinburgh Postnatal Depression Scale (EPDS), Patient Health Questionnaire-9 (PHQ-9), and Generalized Anxiety Disorder-7 (GAD-7). Associations were assessed with χ^2^ tests (categorical comparisons), Pearson correlations, and multivariable linear regression models including center and selected sociodemographic/obstetric covariates. **Results**: Self-esteem showed a strong inverse correlation with postpartum depressive symptoms (RSES–EPDS: r = −0.542 overall; r = −0.537 in Bihor; r = −0.552 in Timiș; all *p* < 0.001). Negative correlations were also observed with anxiety (RSES–GAD-7: r = −0.400; *p* < 0.001) and PHQ-9 depressive severity (r = −0.370; *p* < 0.001). Stratified analyses indicated graded symptom burden across self-esteem categories, with higher EPDS risk proportions among women with moderate/low self-esteem within each center (χ^2^ *p* ≤ 0.039). In adjusted models, EPDS (B = −0.37; *p* < 0.001) and GAD-7 (B = −0.15; *p* = 0.021) remained independently associated with lower RSES, alongside study center (Timiș vs. Bihor: B = −1.08; *p* = 0.043) and educational attainment. **Conclusions**: Lower self-esteem co-occurs with postpartum depressive symptoms and, secondarily, anxiety in Romanian women. While the cross-sectional design precludes causal inference, these robust correlational associations support the potential value of self-esteem assessment for early psychosocial risk identification. Longitudinal research is needed to establish temporal relationships and evaluate whether self-esteem can prospectively predict postpartum mental health outcomes.

## 1. Introduction

Pregnancy and the postpartum period involve rapid biological, psychological, and social changes that may increase women’s vulnerability to mental health disorders [1,2,3,4]. Postpartum depression (PPD), defined as major depressive disorder occurring after childbirth [5], represents a major public health concern due to its adverse short- and long-term effects on maternal and child health [6,7]. Psychiatric disorders are among the leading contributors to maternal mortality in Western countries, with mood and anxiety disorders being particularly prevalent during the perinatal period [8,9,10,11]. Recent estimates indicate that approximately 21% of women experience at least one anxiety disorder during the perinatal period [12], while a meta-analysis reported prevalences of 15.2% for anxiety symptoms during pregnancy and up to 15% for anxiety symptoms postpartum [13]. These conditions can impair maternal functioning, disrupt mother–infant bonding, and negatively affect infant development, highlighting the need for early identification and prevention [6,7].

Self-esteem is a key psychosocial construct reflecting an individual’s global sense of self-worth. Low self-esteem during pregnancy and the postpartum period has been associated with depressive and anxiety symptoms, impaired partner relationships, reduced maternal–infant attachment, and early cessation of breastfeeding [1,14,15]. It has been estimated that 30–70% of women experience a decline in self-esteem following childbirth [14], and low self-esteem has been linked to an increased risk of antenatal and postpartum depression, maladaptive health behaviors, and adverse obstetric outcomes [2,16,17,18]. Pregnancy and postpartum-related bodily changes may further exacerbate negative self-evaluations, with body dissatisfaction frequently reported across all stages of pregnancy and after delivery [1,4,15,19]. The Rosenberg Self-Esteem Scale (RSES) is the most widely used and validated instrument for assessing self-esteem, owing to its brevity, ease of administration, and robust psychometric properties [19,20].

In addition to psychosocial vulnerability, obstetric and neonatal stressors may contribute to postpartum psychological distress. Mothers of infants requiring neonatal intensive care often experience heightened anxiety, depressive symptoms, and difficulties in early attachment due to medical uncertainty, environmental stress, and maternal–infant separation [21,22,23].

Empirical data on postpartum mental health from Eastern Europe, particularly Romania, remain limited. Although theoretical frameworks often conceptualize self-esteem as a vulnerability factor that predisposes individuals to depression (vulnerability model), the relationship may be bidirectional, with depressive episodes also eroding self-esteem over time (scar model). Cross-sectional designs cannot establish temporal precedence or causal directionality; therefore, the present study examines correlational associations between self-esteem and postpartum psychological symptoms while acknowledging that both vulnerability and scar processes may operate simultaneously. The present study aimed to: (1) describe self-esteem levels among postpartum Romanian women, (2) examine the strength and direction of correlational associations between self-esteem and postpartum depression/anxiety symptoms, and (3) test whether these associations remain statistically significant after adjustment for sociodemographic and clinical covariates.

## 2. Materials and Methods

### 2.1. Study Design and Setting

This cross-sectional observational study was conducted in two public maternity hospitals in Western Romania: the Municipal Clinical Emergency Hospital Timișoara (Timiș County) and the Municipal Clinical Hospital “Dr. Gavril Curteanu” Oradea (Bihor County). Participants were recruited during the early postpartum period, while still hospitalized following delivery. Data collection was carried out between 14 October 2024 and 15 November 2025 using structured self-administered questionnaires, supplemented by clinical data extracted from medical records.

### 2.2. Participants and Recruitment

Eligible participants were women 2–4 days postpartum who delivered in one of the two participating hospitals, were clinically stable, and were able to read and complete questionnaires in Romanian. Women with stillbirth, severe obstetric complications precluding participation, or documented severe cognitive impairment were excluded. Consecutive eligible women were approached during their postpartum hospital stay and invited to participate. The final analytic sample consisted of 201 women, including 100 from Bihor County and 101 from Timiș County.

### 2.3. Sociodemographic, Obstetric, and Neonatal Data

Sociodemographic and psychosocial data were collected using structured self-administered questionnaires distributed during the first postpartum period when women were still in hospital. This time point was selected to allow initial physical recovery after childbirth and sufficient maternal–infant stabilization, while minimizing recall bias and capturing early postpartum psychological status. Participants completed the questionnaires independently in a quiet clinical setting, with trained study personnel available to provide clarification if needed. The questionnaire assessed age, place of residence (urban/rural), ethnicity, household composition, educational attainment, employment status and occupational category, marital status, perceived family income level, and housing characteristics (satisfaction with housing, number of rooms, and number of household members).

Obstetric and pregnancy-related data were primarily extracted from medical records and supplemented by maternal report when necessary. These variables included maternal anthropometric measures (height, weight at pregnancy onset and at delivery, gestational weight gain, body mass index), selected lifestyle factors during pregnancy (smoking and alcohol use), pregnancy-related symptoms and complications, and indicators of antenatal care utilization.

Delivery characteristics (mode of delivery, medically induced labor, term versus preterm birth, twin delivery, and documented delivery complications) and neonatal outcomes (gestational age at birth, birth weight and length, Apgar scores at 1 and 5 min, and duration of neonatal intensive care unit [NICU] stay when applicable) were obtained from hospital medical records.

### 2.4. Psychological Measures

#### 2.4.1. Depressive Symptoms

Postpartum depressive symptoms were assessed using the Edinburgh Postnatal Depression Scale (EPDS), a 10-item self-report instrument evaluating symptoms over the preceding seven days [24]. Each item is scored from 0 to 3, yielding a total score range of 0–30, with higher scores indicating greater symptom severity. EPDS scores were analyzed as a continuous variable, and two dichotomous indicators were derived using established cut-offs: EPDS ≥ 10 (possible depression) and EPDS ≥ 13 (probable major depression), based on validated sensitivity and specificity estimates [25].

Depressive symptom severity was additionally assessed using the Patient Health Questionnaire-9 (PHQ-9), which measures nine DSM-based depressive symptoms on a 0–3 scale (total score range: 0–27) [26]. Severity categories were defined using standard cut-offs: minimal (0–4), mild (5–9), moderate (10–19), and severe depression (20–27).

The Romanian version (EPDS-R) has demonstrated excellent internal consistency in validation studies (α = 0.89) [24]. EPDS scores were analyzed both continuously and categorically using two established cut-offs: EPDS ≥ 10 (possible depression; sensitivity = 0.85, specificity = 0.84) and EPDS ≥ 13 (probable major depression; sensitivity = 0.66, specificity = 0.95) [25]. Internal consistency in the current sample was good (α = 0.83; 95% CI: 0.79–0.86).

The PHQ-9 was originally developed by Robert L. Spitzer et al. after receiving a grant funded by Pfizer Inc. (New York, NY, USA) and is freely available for use, reproduction, translation, and distribution without permission. The Romanian version, translated by the instrument’s developers, was obtained from https://www.phqscreeners.com (accessed on 20 January 2026) and is considered the official translated version [27]. Internal consistency for the PHQ-9 was acceptable (Cronbach’s α = 0.82; 95% CI: 0.78–0.86), consistent with previous findings (α = 0.839) [28].

#### 2.4.2. Anxiety Symptoms

Anxiety symptoms were measured using the Generalized Anxiety Disorder 7-item scale (GAD-7) [29]. Each item is rated from 0 to 3, producing total scores ranging from 0 to 21. Anxiety severity was categorized using standard cut-offs: minimal (0–4), mild (5–9), moderate (10–14), and severe anxiety (15–21). The GAD-7 was originally developed by Spitzer et al. and is freely available without permission requirements. The Romanian version, translated by MAPI Research Institute, was obtained from https://www.phqscreeners.com. In the present study, the GAD-7 demonstrated excellent internal consistency (Cronbach’s α = 0.91; 95% CI: 0.89–0.93), consistent with previous validation studies (α = 0.92) [30,31].

#### 2.4.3. Self-Esteem

Self-esteem was measured using the Romanian version of the Rosenberg Self-Esteem Scale [32]. Global self-esteem was assessed using the Rosenberg Self-Esteem Scale (RSES), a widely used and validated 10-item instrument designed to measure overall self-worth [33]. Higher scores indicate greater self-esteem. Each item is rated on a four-point Likert scale, yielding a total score ranging from 0 to 30, with higher scores indicating greater self-esteem. Based on commonly used cut-off values, self-esteem was categorized as low (<15), normal (15–25), or high (>25). Internal consistency in the present sample was good for the Rosenberg Self-Esteem Scale (Cronbach’s α = 0.807).

### 2.5. Data Management and Statistical Analysis

All data were entered into a secure electronic database and checked for completeness and internal consistency. Descriptive statistics were used to summarize sociodemographic, obstetric, neonatal, and psychological variables in the total sample and stratified by county (Bihor vs. Timiș). Continuous variables were reported as means and standard deviations, and categorical variables as frequencies and percentages.

Between-county comparisons were conducted using independent-samples *t*-tests for continuous variables when assumptions were met, and χ^2^ tests or Fisher’s exact tests for categorical variables, as appropriate. Associations between self-esteem and psychological outcomes (EPDS, PHQ-9, and GAD-7 total scores) were examined using Pearson correlation coefficients in the pooled sample and separately by center. Correlation coefficients (r) and corresponding *p*-values were reported. Sample size was determined by consecutive recruitment during the study period (2024–2025) at both participating centers, yielding a final analytic sample of 201 participants. Post hoc power analysis indicated that this sample provides 81% power to detect bivariate correlations of r ≥ 0.20 and between-group differences in d ≥ 0.40 at α = 0.05 (two-tailed). Post hoc verification confirmed that all statistically significant findings (RSES-EPDS correlation r = −0.54) substantially exceeded these thresholds.

Multivariable linear regression was performed to estimate independent associations between self-esteem (RSES) and depressive (EPDS) and anxiety (GAD-7) symptoms after adjusting for potential confounders. RSES was modeled as the dependent variable with EPDS and GAD-7 as primary predictors to facilitate covariate adjustment; this analytical specification does not imply causal direction, as the cross-sectional design is equally consistent with vulnerability models (self-esteem predicting depression/anxiety) and scar models (depression/anxiety eroding self-esteem). The following covariates were included: study center (Bihor vs. Timiș) to account for unmeasured contextual differences; education level as a socioeconomic proxy; reproductive history variables (history of miscarriage/bleeding, pregnancy planning status) as markers of psychological vulnerability; and age at menarche as a developmental indicator. Maternal age, parity, mode of delivery, and neonatal outcomes were excluded based on lack of evidence for confounding in preliminary analyses.

PHQ-9 was not included in the multivariable model due to high correlation with EPDS (r > 0.50), which would introduce multicollinearity. EPDS was selected as the primary measure of depressive symptoms given its specific validation for postpartum populations.

Multicollinearity and autocorrelation diagnostics confirmed model assumptions were met. The Durbin-Watson statistic was 1.96 (*p* = 0.742), indicating no significant autocorrelation. VIF values for all predictors in the final model were well below the conventional threshold of 5: EPDS (VIF = 1.32), GAD-7 (VIF = 1.71), center (VIF = 1.02), education (VIF = 1.03), pregnancy planning (VIF = 1.03), history of miscarriage/bleeding (VIF = 1.02), and age at menarche (VIF = 1.03). These values confirm that multicollinearity did not substantially influence coefficient estimates.

Multicollinearity was assessed using Variance Inflation Factor (VIF) values for all potential predictors. The Durbin-Watson test was used to assess autocorrelation of residuals. PHQ-9 was excluded from the final multivariable model due to conceptual redundancy with EPDS (both measure depressive symptoms), although VIF diagnostics indicated that statistical multicollinearity would not preclude its inclusion. EPDS was retained as the primary depression measure given its specific validation and widespread use in postpartum populations.

Even with high bivariate correlations between psychological measures (r = 0.64–0.79), VIF values remained low (all < 2.0), indicating acceptable collinearity levels for regression modeling.

A directed acyclic graph (DAG) was constructed to explicate assumed causal relationships between self-esteem, postpartum mental health outcomes, and potential confounders. The DAG guided a priori covariate selection based on theoretical plausibility and identification of confounding pathways, while acknowledging unmeasured factors such as social support as sources of residual bias.

All statistical analyses were performed using RStudio software version 4.3.3 (Posit Software, PBC, Boston, MA, USA), applying complete-case analyses for each variable. A two-sided *p*-value < 0.05 was considered statistically significant.

### 2.6. Ethical Considerations

The study protocol was reviewed and approved by the institutional ethics committees of both participating hospitals. Ethical approval was granted by the Ethics Committee of the Municipal Clinical Emergency Hospital Timișoara (approval no. E-5652/14.10.2024), authorizing access to obstetric and neonatal medical records within Obstetrics–Gynecology Clinic IV, and by the Ethics Committee of the Clinical County Emergency Hospital Bihor (approval no. 14136/08.05.2025), permitting access to institutional databases for doctoral research purposes. All participants received written and verbal information about the study and provided written informed consent prior to participation. Participation was voluntary, and all data were anonymized prior to analysis to ensure confidentiality.

## 3. Results

Of 201 participants included in the analysis (Bihor, n = 100; Timiș, n = 101), the mean (SD) maternal age was 29.7 (5.8) years. Overall, 43.8% of women resided in rural areas, 93.0% were of Romanian ethnicity, and 57.2% had completed higher education. The majority of participants lived with a husband and children (65.3%), were married (89.1%), reported a stressful job (59.4%), and perceived their family income as adequate (74.6%). There were no significant differences between the 2 counties regarding any baseline sociodemographic or obstetric characteristics (*p* > 0.05) (Table 1).

Mean (SD) gestational weight gain was higher among women in Bihor compared with Timiș (17.1 [5.6] kg vs. 15.4 [5.4] kg; *p* = 0.049). Neonates in Timiș had significantly higher mean Apgar scores at 1 min (9.1 [1.0] vs. 8.7 [1.1]; *p* = 0.03) and 5 min (9.4 [0.8] vs. 9.0 [0.5]; *p* = 0.002). No significant differences were observed between centers regarding pre-pregnancy mean BMI, gestational age at birth, birth weight, birth length, or duration of neonatal intensive care unit stay (all *p* > 0.05) (Table 2).

Cesarean delivery was the predominant mode of birth (74.6%), with similar proportions in Bihor (75.0%) and Timiș (74.3%). Medically induced labor (8.5%), preterm birth (15.1%), twin pregnancies (3.5%), and delivery complications (7.5%) were comparable across centers. No statistically significant differences were observed between the 2 hospitals for any obstetric or delivery characteristics (all *p* > 0.05) (Table 3).

The distribution of depressive and anxiety symptoms differed significantly across self-esteem categories in the overall sample and across study centers (Table 4).

For the EPDS ≥ 10 threshold, a significant association with self-esteem was observed both overall (*p* < 0.001) and within each study center (Bihor: *p* = 0.039; Timiș: *p* < 0.001). In the overall sample, the proportion of women screening positive increased markedly with decreasing self-esteem, from 11.8% among those with high self-esteem to 42.2% among those with normal self-esteem and 80.0% among those with low self-esteem. A similar gradient was observed in both centers. In Bihor, 17.1% of women with high self-esteem screened positive compared with 38.7% of those with normal self-esteem and 66.7% of those with low self-esteem. In Timiș, corresponding proportions were 6.1%, 45.5%, and 100.0%, respectively.

For the more stringent EPDS ≥13 threshold, the association with self-esteem remained significant in the overall sample (*p* < 0.001) and in Timiș (*p* < 0.001), but did not reach statistical significance in Bihor (*p* = 0.161). Overall, EPDS ≥ 13 positivity increased from 4.4% in women with high self-esteem to 19.5% in those with normal self-esteem and 60.0% in those with low self-esteem. In Bihor, although a similar numerical trend was observed, the small size of the low self-esteem subgroup likely limited statistical power.

PHQ-9 symptom severity differed significantly by self-esteem level in the overall sample (*p* < 0.001) and in Bihor (*p* < 0.001), whereas no statistically significant association was observed in Timiș (*p* = 0.244). Across the overall sample and in Bihor, high self-esteem was predominantly associated with minimal depressive symptoms, while moderate and severe symptom categories were more frequent among women with lower self-esteem.

Similarly, GAD-7 anxiety severity showed a significant association with self-esteem both overall (*p* < 0.001) and within each center (Bihor: *p* = 0.007; Timiș: *p* = 0.007). Women with lower self-esteem exhibited substantially higher proportions of moderate-to-severe anxiety symptoms compared with those reporting high self-esteem, a pattern that was consistent across centers despite small subgroup sizes.

**Figure 1:**
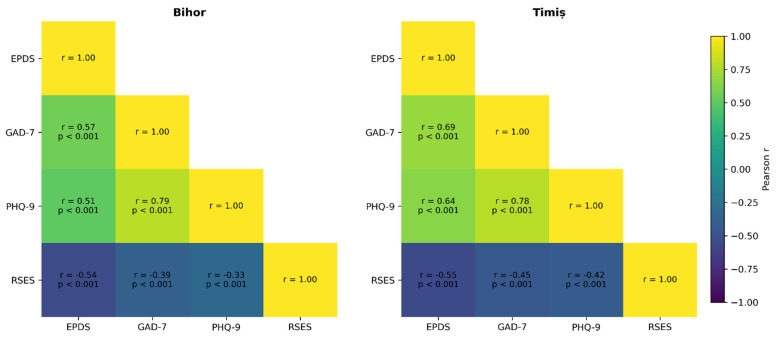
Correlation matrices illustrating the relationships between depressive symptoms (EPDS, PHQ-9), anxiety symptoms (GAD-7), and global self-esteem (RSES) in the Bihor and Timiș cohorts.

Figure 1 illustrates Pearson correlation matrices between depressive symptoms (EPDS and PHQ-9), anxiety symptoms (GAD-7), and self-esteem (RSES), stratified by study center (Bihor and Timiș). In both centers, measures of depressive and anxiety symptoms were strongly and positively intercorrelated. In Bihor, EPDS was moderately correlated with GAD-7 (*r* = 0.57, *p* < 0.001) and PHQ-9 (*r* = 0.51, *p* < 0.001), while the association between GAD-7 and PHQ-9 was particularly strong (*r* = 0.79, *p* < 0.001). A comparable pattern was observed in Timiș, where EPDS correlated strongly with GAD-7 (*r* = 0.69, *p* < 0.001) and PHQ-9 (*r* = 0.64, *p* < 0.001), and GAD-7 and PHQ-9 again showed a high correlation (*r* = 0.78, *p* < 0.001).

Self-esteem demonstrated consistent moderate inverse associations with depressive and anxiety symptoms across both centers. In Bihor, RSES scores were negatively correlated with EPDS (*r* = −0.54, *p* < 0.001), GAD-7 (*r* = −0.39, *p* < 0.001), and PHQ-9 (*r* = −0.33, *p* < 0.001). Similar inverse relationships were observed in Timiș, with correlations of *r* = −0.55 for EPDS, *r* = −0.45 for GAD-7, and *r* = −0.42 for PHQ-9 (all *p* < 0.001).

**Figure 2:**
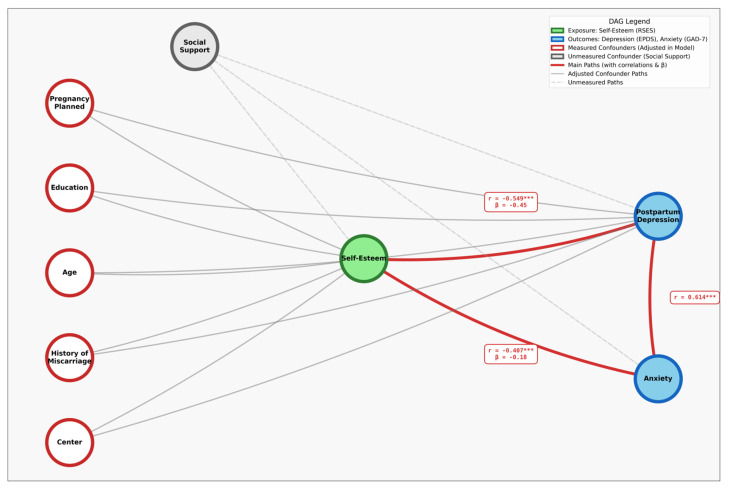
Directed acyclic graph (DAG) illustrating assumed causal relationships between self-esteem and postpartum mental health outcomes. *** indicates p < 0.001.

The observed bivariate and adjusted associations were consistent with the causal structure hypothesized in the DAG (Figure 2), though unmeasured confounding by social support precludes definitive causal inference.

In multivariable linear regression with RSES total score as the dependent variable (Table 5), the overall model was statistically significant and explained 36.8% of the variance in postpartum self-esteem (R = 0.606, R^2^ = 0.368, adjusted R^2^ = 0.337, *p* < 0.001). Specifically, EPDS total score showed a robust inverse association with RSES (B = −0.37, SE = 0.06, 95% CI: −0.50 to −0.25; *p* < 0.001; standardized β = −0.45), indicating that each one-point increase in EPDS corresponded to a 0.37-point decrease in self-esteem after adjustment for covariates. Anxiety symptoms were also inversely related to self-esteem (GAD-7: B = −0.15, SE = 0.07, 95% CI: −0.29 to −0.02; *p* = 0.021; β = −0.18).

A significant center effect was observed, with participants recruited in Timiș exhibiting lower RSES scores compared with Bihor (B = −1.08, SE = 0.53, 95% CI: −2.12 to −0.03; *p* = 0.043; β = −0.25). Educational attainment was positively associated with self-esteem relative to the reference category (middle school), with higher RSES scores among women with high school (B = 4.52; *p* = 0.015), university education (B = 4.95; *p* = 0.007), and primary school (B = 4.56; *p* = 0.041). Pregnancy planning was not significantly associated with RSES (B = −0.52; *p* = 0.339). A history of miscarriage/bleeding was associated with lower self-esteem (B = −1.69, 95% CI: −3.22 to −0.16; *p* = 0.030), and older age at menarche was also modestly associated with lower RSES (B = −0.36 per year; *p* = 0.044).

## 4. Discussion

In this cross-sectional study of 201 postpartum women recruited from two public maternity hospitals in Western Romania (Bihor and Timiș), self-esteem emerged as a salient psychosocial correlate of postpartum psychological distress. Across the pooled sample, higher Rosenberg Self-Esteem Scale (RSES) scores were consistently associated with lower depressive symptom burden, with a strong inverse correlation between RSES and EPDS total score (r ≈ −0.54), replicated within each center. Parallel associations were observed for anxiety and general depressive symptom severity: RSES correlated negatively with GAD-7 (r ≈ −0.40) and PHQ-9 (r ≈ −0.37), again showing comparable direction and magnitude across counties.

Longitudinal evidence from Korean postpartum women followed across eight years demonstrated bidirectional relationships between depression and self-esteem, with each influencing the other over time, and these dynamics were moderated by gestational weight gain patterns [14].

An important interpretive consideration is that the cross-sectional design precludes determination of causal directionality. While the observed inverse associations between self-esteem and depressive symptoms are consistent with cognitive vulnerability models positing self-esteem as a predisposing factor, they are equally consistent with scar models in which depressive symptoms erode self-worth. Indeed, bidirectional relationships are plausible: low self-esteem may increase vulnerability to postpartum depression, while depressive episodes may further diminish self-esteem, creating a reinforcing cycle. Longitudinal designs with multiple assessment points are necessary to disentangle temporal ordering and test competing directional hypotheses.

Clinically meaningful gradients were also evident when outcomes were stratified by self-esteem category. In both Bihor and Timiș, women classified as having high self-esteem showed substantially lower proportions meeting EPDS risk thresholds (≥10 and ≥13) and were more likely to fall within minimal symptom categories on PHQ-9 and GAD-7. Conversely, the low self-esteem subgroup although small showed concentrated symptom burden, with most participants meeting clinically relevant thresholds.

In multivariable analyses, the regression model explained 36.8% of variance in postpartum self-esteem (R^2^ = 0.368, *p* < 0.001), indicating that depressive symptoms, anxiety symptoms, and measured sociodemographic and reproductive factors account for a substantial proportion of self-esteem variability. This magnitude of explained variance is consistent with prior psychosocial research in perinatal populations. EPDS total score demonstrated a stable inverse association with RSES in regression modeling, indicating that each incremental increase in depressive symptom severity corresponded to lower self-esteem after accounting for other covariates. Anxiety symptoms showed a smaller but statistically significant association with self-esteem in the adjusted model, suggesting that self-esteem is relevant to anxiety as well, though the relationship appeared less pronounced than for depressive symptomatology. In addition, contextual and sociodemographic factors contributed: self-esteem differed by center (lower RSES in Timiș relative to Bihor in the adjusted model), and educational attainment was positively associated with self-esteem, while a history of miscarriage/bleeding and age at menarche showed smaller negative associations.

The early postpartum period represents a phase of pronounced psychological vulnerability, characterized by rapid role transitions, physical recovery, sleep disruption, and substantial psychosocial adjustment demands. Systematic reviews have consistently identified this period as one of heightened risk for the onset or exacerbation of postpartum depressive symptoms, reflecting the cumulative burden of biological, emotional, and social stressors faced by new mothers [34]. Cross-sectional evidence from 1423 Brazilian women indicated that self-esteem remained stable across the three gestational trimesters but declined significantly in the postpartum period, paralleling increased depressive symptoms and decreased body appreciation after delivery [35].

Within this context, cognitive vulnerability factors particularly low self-esteem and heightened self-criticism may increase susceptibility to maladaptive cognitive schemas, such as perceived inadequacy and negative self-appraisal, which are central to depressive symptom development and maintenance.

Empirical evidence supports this cognitive vulnerability framework. In a large cross-sectional study of 686 postpartum women, elevated self-criticism was strongly associated with increased negative automatic thoughts and greater depressive symptom severity, with these cognitive processes accounting for a meaningful proportion of variance in depressive outcomes [36]. Similarly, a study of 550 mothers of infants under 24 months demonstrated that self-critical dimensions (inadequate-self and hated-self) were associated with postpartum depressive symptoms and mediated the relationship between a maternal history of depression and mother–infant bonding difficulties [37]. These findings align closely with the strong inverse association observed between self-esteem and EPDS scores in the present sample, reinforcing the interpretation that self-esteem-related cognitive processes play a central role in postpartum depressive symptomatology. Although self-esteem was significantly associated with both depressive and anxiety symptoms in the present study, the relationship with depression was consistently stronger than that observed for anxiety. This pattern suggests that self-esteem may be more proximally linked to depressive cognitions than to generalized anxiety symptomatology. Clinically, this distinction is plausible: depressive syndromes explicitly incorporate negative self-evaluation, diminished self-worth, and feelings of failure, whereas anxiety disorders are more strongly driven by excessive worry, hypervigilance, and threat anticipation.

This differential pattern is supported by longitudinal evidence. A large meta-analysis synthesizing 77 longitudinal studies on depression and 18 on anxiety demonstrated that low self-esteem more robustly predicts subsequent depressive symptoms than anxiety symptoms, supporting a vulnerability model in which negative self-evaluation plays a particularly central role in depression [38]. Nevertheless, postpartum-specific clinical studies indicate that self-critical processes also relate to anxiety symptoms. For example, a clinical comparison of postpartum women with and without depression showed that depressed mothers exhibited higher levels of self-criticism, which were also positively associated with state anxiety, consistent with a transdiagnostic role for self-evaluative vulnerability across internalizing disorders [39].

Accordingly, the observed inverse associations between RSES and both GAD-7 and PHQ-9 scores in the present study suggest that self-esteem may function as a shared vulnerability factor contributing to broader internalizing symptom profiles in the postpartum period, while remaining more strongly linked to depressive symptomatology.

Importantly, the categorical analyses reinforce the clinical relevance of these associations. Across both maternity centers, women with moderate or low self-esteem were substantially more likely to screen positive on EPDS thresholds, and symptom severity distributions on PHQ-9 and GAD-7 shifted in a graded, dose–response manner across self-esteem levels. Such graded associations suggest that self-esteem may not only differentiate women with and without clinically significant symptoms, but may also capture subthreshold vulnerability states. This finding supports the potential utility of incorporating self-esteem assessment into early postpartum screening frameworks to identify women at heightened risk before symptoms escalate to more severe or impairing levels. The present findings align closely with international research identifying self-esteem as a key psychosocial correlate of postpartum depression. In a Saudi Arabian cross-sectional study, postpartum depression severity was strongly and negatively associated with self-esteem (r = −0.63) and perceived social support, with effect sizes comparable to those observed in the Romanian cohort despite substantial cultural and healthcare-system differences [40]. These convergent findings underscore the robustness and cross-cultural generalizability of the relationship between low self-esteem and postpartum depressive symptoms.

Prospective evidence further supports a vulnerability-based interpretation. In a longitudinal study of women without prior clinical depression, instability in self-esteem during pregnancy rather than absolute self-esteem level predicted depressive symptoms at 12 weeks postpartum, independent of baseline mood, age, and socioeconomic status [41].

The pregnancy-to-postpartum transition encompasses multiple domains of potential self-esteem threat. During pregnancy, women experience progressive bodily changes that challenge pre-pregnancy self-concepts, including weight gain, altered body proportions, reduced physical capacity, and perceived loss of bodily autonomy.

The observed association between study center and self-esteem indicating lower adjusted RSES scores in Timiș compared with Bihor should be interpreted cautiously. Center effects likely reflect a constellation of unmeasured contextual factors rather than a direct institutional influence, including differences in patient case-mix, urbanicity, family structures, and social support environments.

Romania exhibits marked regional disparities in healthcare access, with significant variations in the geographic distribution of medical personnel, hospital infrastructure, and quality of care across development regions. Healthcare workforce shortages in rural areas remain critical, as 90% of physicians and nurses are located in urban healthcare settings, resulting in stark regional disparities where some hospitals cannot recruit essential specialists while urban areas remain saturated with medical professionals [42,43]. The Romanian healthcare system struggles with issues such as inadequate infrastructure, regional disparities, a shortage of healthcare professionals, and limited funding, with accessibility of healthcare services particularly hindered in outlying regions [29].

Mental health services show particularly pronounced regional variation. Community mental health services are currently available in only a few regions of the country, and even fewer centers efficiently communicate with other mental health organizations, resulting in discrimination against people with mental illness in relation to access to care [29]. Regions with lower socioeconomic status should be a priority focus as mental illness is frequent in these groups while knowledge is poor and perceived stigma and discrimination is higher compared to other groups [44].

These systemic regional differences may contribute to differential psychosocial vulnerability among postpartum women across counties, potentially mediated through reduced access to preventive mental health services, limited social support infrastructure, or heightened perception of healthcare system inadequacy factors that could erode self-esteem independent of individual educational or economic status.

Descriptive differences in household composition and educational attainment between counties suggest that broader social context may shape self-esteem and psychological vulnerability in meaningful ways. Socioeconomic status—encompassing material capital (household income), human capital (educational attainment), and social capital (occupational prestige)—influences postpartum mental health through multiple pathways including healthcare access, social support, and resource adequacy [45,46]. Neighborhood-level socioeconomic characteristics demonstrate statistically significant associations with postpartum depression risk across populations [47].

Educational attainment showed a consistent positive association with self-esteem, potentially reflecting access to psychosocial resources such as health literacy, perceived control, and problem-solving skills. While perceived income did not differ significantly between regions, education may capture latent socioeconomic gradients not fully reflected by subjective income measures. Prior studies suggest that self-esteem and education may influence postpartum depression through partially distinct pathways, with self-esteem acting as a proximal psychological vulnerability and education functioning as a broader contextual risk marker [48].

Finally, the association between a history of miscarriage or bleeding and lower self-esteem highlights the enduring psychological impact of prior reproductive adversity. Such experiences may contribute to persistent vulnerability, heightened threat appraisal, and reduced self-worth during subsequent pregnancies and the postpartum period. Although this factor was not directly modeled as a determinant of depressive symptoms in the present analyses, it underscores the importance of incorporating reproductive history into comprehensive postpartum psychosocial risk assessments.

## 5. Strengths and Limitations

Several strengths of this study should be acknowledged. The inclusion of two independent maternity centers enhances the generalizability of the findings within Western Romania and allows for internal replication of observed associations. The use of well-validated, internationally recognized instruments (RSES, EPDS, PHQ-9, and GAD-7) strengthens measurement validity and facilitates comparison with prior literature. In addition, the integration of both categorical and continuous analytical approaches provides a nuanced understanding of the relationship between self-esteem and postpartum psychological outcomes.

Nevertheless, important limitations warrant consideration. The cross-sectional design precludes causal inference and determination of temporal precedence. It is not possible to determine whether: (1) low self-esteem predisposes women to postpartum depressive and anxiety symptoms (vulnerability model), (2) psychological distress erodes self-esteem (scar model), or (3) both processes operate bidirectionally in a reinforcing cycle. Although we modeled self-esteem as the dependent variable in regression analyses for statistical purposes, this should not be interpreted as implying directionality. The observed associations are consistent with multiple causal pathways that cannot be distinguished with cross-sectional data. We modeled self-esteem as the dependent variable in regression analyses for analytical convenience to facilitate covariate adjustment; however, this specification does not imply directionality and does not constitute a test of vulnerability or scar hypotheses. The observed associations are consistent with multiple causal pathways that cannot be distinguished with cross-sectional data. Longitudinal designs with repeated measurements are essential to establish temporal ordering and test directional hypotheses.

Longitudinal designs are needed to clarify temporal ordering and causal mechanisms. The relatively small size of the low self-esteem subgroup limits statistical precision for this category and necessitates cautious interpretation of extreme proportions.

Sample size was determined by consecutive recruitment, yielding 201 participants with 81% power to detect moderate correlations (r ≥ 0.20) and group differences (d ≥ 0.40) at α = 0.05. All statistically significant continuous findings substantially exceeded these thresholds, including the primary self-esteem–depression correlation (r = −0.54, *p* < 0.001) which replicated across both centers. However, categorical analyses stratified by self-esteem level (Table 4) were underpowered due to low prevalence of severely diminished self-esteem (n = 5; 2.5% of sample). With such small cell sizes, reported proportions (e.g., 80% = 4/5) are statistically unstable and carry wide confidence intervals, requiring interpretation as preliminary descriptive patterns rather than precise estimates.

Furthermore, although multiple sociodemographic and obstetric variables were included, residual confounding by unmeasured factors such as perceived social support, partner relationship quality, or prior trauma cannot be excluded.

Finally, data were collected during the early postpartum hospitalization period, which may reflect acute emotional and physical recovery processes. Psychological symptom levels and self-esteem may evolve substantially in the weeks and months following discharge, underscoring the need for follow-up assessments.

## 6. Conclusions

In this two-center cross-sectional study of postpartum women in Romania, self-esteem emerged as a salient psychosocial correlate of postpartum depressive and anxiety symptoms. Lower self-esteem was consistently associated with greater depressive symptom burden, showed moderate inverse associations with anxiety and general depressive severity, and remained independently related to psychological distress in multivariable analyses. These associations were robust across centers and were evident both dimensionally and categorically.

While these findings are consistent with self-esteem functioning as a vulnerability factor for postpartum psychological distress, the cross-sectional design precludes causal inference. The observed associations should be interpreted as hypothesis-generating rather than implementation-ready. Longitudinal research is essential to establish temporal precedence and determine whether low self-esteem prospectively predicts postpartum mental health deterioration or whether depressive symptoms erode self-esteem. Interventional trials are needed to evaluate whether self-esteem-targeted interventions can prevent or reduce postpartum depression and anxiety. Only with such prospective and experimental evidence can recommendations be made regarding the clinical utility of self-esteem assessment in postpartum screening or the implementation of self-esteem-focused interventions.

## Figures and Tables

**Table 1:** Sociodemographic and obstetric characteristics of the study population by center.

Variable	Level	Bihor (N = 100)	Timiș (N = 101)	Total (N = 201)	*p* Value
Age at delivery					0.388
	Mean (SD), years	30.1 (6.0)	29.4 (5.6)	29.7 (5.8)	
	Range	14–53	16–41	14–53	
Living environment	Rural	37 (37.0%)	51 (50.5%)	88 (43.8%)	0.054
	Urban	63 (63.0%)	50 (49.5%)	113 (56.2%)	
Ethnicity	Romanian	90 (90.0%)	97 (96.0%)	187 (93.0%)	0.093
	Other	10 (10.0%)	4 (4.0%)	14 (7.0%)	
Living arrangement	N missing	6	2	8	0.848
	Husband + children (±others)	62 (66.0%)	64 (64.6%)	126 (65.3%)	
	Husband only	32 (34.0%)	35 (35.4%)	67 (34.7%)	
Education level	Higher education	62 (62.0%)	53 (52.5%)	115 (57.2%)	0.356
	High school	33 (33.0%)	40 (39.6%)	73 (36.3%)	
	≤Middle school	5 (5.0%)	8 (7.9%)	13 (6.5%)	
Stressful job	N missing	12	19	31	0.140
	Yes	57 (64.8%)	44 (53.7%)	101 (59.4%)	
	No	31 (35.2%)	38 (46.3%)	69 (40.6%)	
Marital status	Married	91 (91.0%)	88 (87.1%)	179 (89.1%)	0.379
	Not married	9 (9.0%)	13 (12.9%)	22 (10.9%)	
Perceived family income	Adequate	70 (70.0%)	80 (79.2%)	150 (74.6%)	0.134
	Inadequate	30 (30.0%)	21 (20.8%)	51 (25.4%)	

**Table 2:** Maternal Anthropometrics and Neonatal Outcomes by County.

Variable	Missing (N)		Bihor (N = 100)	Timiș (N = 101)	Total (N = 201)	*p*-Value
BMI pregnancy onset (kg/m^2^)	17		24.0 (4.6)	23.2 (4.0)	23.6 (4.3)	0.2362
BMI pregnancy onset	17	Underweight	8.0 (9.6%)	7.0 (7.1%)	15.0 (8.2%)	0.1871
		Normal weight	42.0 (50.6%)	65.0 (65.7%)	107.0 (58.8%)	
		Overweight	26.0 (31.3%)	19.0 (19.2%)	45.0 (24.7%)	
		Obesity	7.0 (8.4%)	8.0 (8.1%)	15.0 (8.2%)	
Gestational weight gain (kg)	25					0.049
Mean (SD)	—		17.1 (5.6)	15.4 (5.4)	16.1 (5.6)	
Gestational age at birth (weeks)	6		37.5 (4.4)	38.0 (3.8)	37.8 (4.1)	0.421
Birth weight (g)	4		3201.8 (602.6)	3193.4 (506.5)	3197.5 (554.0)	0.916
Birth length (cm)	4		51.6 (5.2)	50.9 (2.6)	51.2 (4.1)	0.227
Apgar score at 1 min	9		8.7 (1.1)	9.1 (1.0)	8.9 (1.1)	0.026
Apgar score at 5 min	33		9.0 (0.5)	9.4 (0.8)	9.2 (0.7)	0.002
Neonatal intensive care unit stay (days)	165		8.5 (11.8)	6.2 (4.5)	7.6 (9.6)	0.410

**Table 3:** Sociodemographic, obstetric, and neonatal profile stratified by study center.

Variable	Category	Missing (n)	Total (N = 201)	Bihor (N = 100)	Timiș (N = 101)	*p*-Value
Mode of delivery, n (%)	Cesarean section	0	150 (74.6)	75 (75.0)	75 (74.3)	1.000
	Vaginal delivery		51 (25.4)	25 (25.0)	26 (25.7)	
Medically induced labor, n (%)	Yes	1	17 (8.5)	10 (10.1)	7 (6.9)	0.582
	No		183 (91.5)	89 (89.9)	94 (93.1)	
Term birth, n (%)	Yes	2	155 (77.9)	76 (77.6)	79 (78.2)	1.000
	No		44 (22.1)	22 (22.4)	22 (21.8)	
Preterm birth, n (%)	Yes	2	22 (10.9%)	10 (9.9%)	12 (12.0%)	0.914
	No		179(89.1%)	91 (90.1%)	88 (88.0%)	
Twin pregnancy, n (%)	Yes	3	7 (3.5)	4 (4.1)	3 (3.0)	0.720
	No		191 (96.5)	94 (95.9)	97 (97.0)	
Delivery complications, n (%)	Yes	1	15 (7.5)	6 (6.1)	9 (8.9)	0.619
	No		185 (92.5)	93 (93.9)	92 (91.1)	

Notes: Data are presented as n (%). *p*-values refer to comparisons between centers and were calculated using χ^2^ tests or Fisher’s exact tests, as appropriate. Percentages are calculated based on available data.

**Table 4:** Distribution of depressive and anxiety outcomes by self-esteem level and study center.

	Overall						Bihor					Timis				
	Category	High (N = 68)	Low (N = 5)	Normal (N = 128)	Total (N = 201)	*p*	High (N = 35)	Low (N = 3)	Normal (N = 62)	Total (N = 100)	*p*	High (N = 33)	Low (N = 2)	Normal (N = 66)	Total (N = 101)	*p*
EPDS risk ≥ 10	No	60 (88.2)	1 (20.0)	74 (57.8)	135 (67.2)	<0.001	29 (82.9)	1 (33.3)	38 (61.3)	68 (68.0)	0.039	31 (93.9)	0 (0.0)	36 (54.5)	67 (66.3)	<0.001
	Yes	8 (11.8)	4 (80.0)	54 (42.2)	66 (32.8)		6 (17.1)	2 (66.7)	24 (38.7)	32 (32.0)		2 (6.1)	2 (100.0)	30 (45.5)	34 (33.7)	
EPDS risk ≥ 13	No	65 (95.6)	2 (40.0)	103 (80.5)	170 (84.6)	<0.001	33 (94.3)	2 (66.7)	51 (82.3)	86 (86.0)	0.161	32 (97.0)	0 (0.0)	52 (78.8)	84 (83.2)	<0.001
	Yes	3 (4.4)	3 (60.0)	25 (19.5)	31 (15.4)		2 (5.7)	1 (33.3)	11 (17.7)	14 (14.0)		1 (3.0)	2 (100.0)	14 (21.2)	17 (16.8)	
PHQ-9 severity level	Minimal	40 (58.8)	2 (40.0)	57 (44.5)	99 (49.3)	<0.001	17 (48.6)	1 (33.3)	25 (40.3)	43 (43.0)	<0.001	23 (69.7)	1 (50.0)	32 (48.5)	56 (55.4)	0.244
	Mild	23 (33.8)	1 (20.0)	49 (38.3)	73 (36.3)		13 (37.1)	0 (0.0)	26 (41.9)	39 (39.0)		10 (30.3)	1 (50.0)	23 (34.8)	34 (33.7)	
	Moderate	5 (7.4)	1 (20.0)	21 (16.4)	27 (13.4)		5 (14.3)	1 (33.3)	11 (17.7)	17 (17.0)		0 (0.0)	0 (0.0)	10 (15.2)	10 (9.9)	
	Severe	0 (0.0)	1 (20.0)	1 (0.8)	2 (1.0)		0 (0.0)	1 (33.3)	0 (0.0)	1 (1.0)		0 (0.0)	0 (0.0)	1 (1.5)	1 (1.0)	
GAD-7 severity level	Minimal	49 (72.1)	1 (20.0)	62 (48.4)	112 (55.7)	<0.001	23 (65.7)	0 (0.0)	28 (45.2)	51 (51.0)	0.007	26 (78.8)	1 (50.0)	34 (51.5)	61 (60.4)	0.007
	Mild	15 (22.1)	0 (0.0)	42 (32.8)	57 (28.4)		8 (22.9)	0 (0.0)	20 (32.3)	28 (28.0)		7 (21.2)	0 (0.0)	22 (33.3)	29 (28.7)	
	Moderate	2 (2.9)	2 (40.0)	12 (9.4)	16 (8.0)		2 (5.7)	2 (66.7)	6 (9.7)	10 (10.0)		0 (0.0)	0 (0.0)	6 (9.1)	6 (5.9)	
	Severe	2 (2.9)	2 (40.0)	12 (9.4)	16 (8.0)		2 (5.7)	1 (33.3)	8 (12.9)	11 (11.0)		0 (0.0)	1 (50.0)	4 (6.1)	5 (5.0)	

Notes: Data are presented as n (column %). Pearson’s chi-squared test was used to assess associations between self-esteem level and each psychological outcome within the overall sample and within each study center. EPDS = Edinburgh Postnatal Depression Scale; PHQ-9 = Patient Health Questionnaire-9; GAD-7 = Generalized Anxiety Disorder-7.

**Table 5:** Multivariable linear regression predicting Rosenberg Self-Esteem Scale (RSES) total score in postpartum women (N = 201).

Predictor	B	SE	95% CI for B	t	*p*	Standardized β	95% CI for β
Intercept	38.84	2.88	33.16, 44.51	13.51	<0.001	—	—
EPDS total score	−0.37	0.06	−0.50, −0.25	−5.96	<0.001	−0.45	−0.60, −0.30
GAD-7 total score	−0.15	0.07	−0.29, −0.02	−2.33	0.021	−0.18	−0.33, −0.03
Center							
Timiș vs. Bihor	−1.08	0.53	−2.12, −0.03	−2.03	0.043	−0.25	−0.49, −0.01
Education level							
High school vs. middle school	4.52	1.85	0.87, 8.17	2.44	0.015	1.04	0.20, 1.89
University vs. middle school	4.95	1.82	1.36, 8.53	2.72	0.007	1.14	0.31, 1.97
Primary school vs. middle school	4.56	2.22	0.19, 8.94	2.06	0.041	1.05	0.04, 2.06
Pregnancy planned (No vs. Yes)	−0.52	0.54	−1.60, 0.55	−0.96	0.339	−0.12	−0.37, 0.13
History of miscarriage/bleeding (No vs. Yes)	−1.69	0.77	−3.22, −0.16	−2.19	0.030	−0.39	−0.74, −0.04
Age at menarche (years)	−0.36	0.18	−0.70, −0.01	−2.03	0.044	−0.12	−0.24, −0.00

B = unstandardized regression coefficient; SE = standard error; CI = confidence interval; β = standardized regression coefficient. Reference categories: Bihor (center), middle school (education), planned pregnancy = yes, miscarriage/bleeding = yes. EPDS = Edinburgh Postnatal Depression Scale; GAD-7 = Generalized Anxiety Disorder Scale.

## Data Availability

The data presented in this study are available from the corresponding author upon reasonable request. The data are not publicly available due to privacy and ethical restrictions related to sensitive participant information.
